# More on how and why: a response to commentaries

**DOI:** 10.1007/s10539-013-9380-4

**Published:** 2013-05-22

**Authors:** Kevin N. Laland, John Odling-Smee, William Hoppitt, Tobias Uller

**Affiliations:** 1 School of Biology, University of St Andrews, St Andrews, UK; 2 Department of Life Sciences, Anglia Ruskin University, Cambridge, UK; 3 Mansfield College, University of Oxford, Oxford, UK; 4 Department of Zoology, Edward Grey Institute, University of Oxford, Oxford, UK

**Keywords:** Proximate causation, Ultimate causation, Mayr, Niche construction, Developmental plasticity, Developmental bias, Extended evolutionary synthesis

## Abstract

We are grateful to the commentators for taking the time to respond to our article. Too many interesting and important points have been raised for us to tackle them all in this response, and so in the below we have sought to draw out the major themes. These include problems with both the term ‘ultimate causation’ and the proximate-ultimate causation dichotomy more generally, clarification of the meaning of reciprocal causation, discussion of issues related to the nature of development and phenotypic plasticity and their roles in evolution, and consideration of the need for an extended evolutionary synthesis.

## Problems with the term ‘ultimate causation’

We have argued that Mayr’s proximate-ultimate causation dichotomy is problematic. Clear distinctions between cause, function and evolutionary history are important, but these can be made without Mayr’s terminology, which we feel is ambiguous, dated, divisive and discourages full consideration of the role of development in evolution.

That current use of the term ‘ultimate causation’ is confused is beautifully illustrated by the commentaries. For Dickins and Barton ultimate causation “is the exposure of *function*”. Gardner writes that “Mayr gave a firmly *historical* interpretation of ultimate explanations” (but then takes issue with this, and recasts ultimate causation as “adaptive rationale”). Watt reads it as evolutionary *history* (but claims it “morphs into” mechanism). Calcott views proximate-ultimate as “a false dichotomy”, whilst for Haig it has different meanings depending on whether one is critic or advocate, Mayr himself or modern user. The two commentaries that defend “ultimate causation”, do not agree on what it is. Dickins and Barton believe “ultimate accounts…potentially include…selectively neutral traits spread by genetic drift”, whilst Gardner writes that it is “incorrect” to allow random drift to form the basis of ultimate explanations. Haig admits Mayr was “not particularly clear”, Gardner characterizes him as “sloppy”, and cites Ariew who describes Mayr “unclear in his own mind”. This confusion has been a feature of the literature since 1961, with various authors interpreting Mayr’s “ultimate causation” as evolutionary history (Beatty 1994; Dewsbury 1999; Ariew 2003; Amundson 2005), as function (Francis 1990; Hogan 1994; Scott-Phillips et al. 2011) and as both (Sherman 1988; MacDougall-Shackleton 2011). No wonder Ariew (2003, p. 553) asks “Why don’t people get it?”.

The answer to Ariew’s question is straight-forward. The term “ultimate causation”, when presented as an answer to Mayr’s “why?” question, is inherently ambiguous. “Why?” implies *function*—what is the character for? Thus in discussing ultimate causes, Mayr (1974, p. 108) writes:

It is necessary for the completion of a causal analysis to ask for any feature, why it exists, that is what its function and role in the life of the particular organism is.

Yet functions are not causes. Functions are descriptions of what characters are fashioned to do. The bird migrates *in order to* get better access to food or mates, but, as many previous researchers have pointed out, the outcome of a behavior cannot determine its occurrence (Francis 1990; Hogan 1994; Ariew 2003; Thierry 2005). Rather, historical events have led to the evolution of proximate causes of the migratory behaviour by selecting for heritable variation that predisposes birds to respond to appropriate cues with migration. Hence, the use of “cause” in “ultimate cause” implies that it is evolutionary history that Mayr has in mind, and other statements of Mayr’s back this up:

These are causes that have a history and that have been incorporated into the system through many thousands of generations of natural selection (1961, p. 1503)

Ultimate causation means a causation responsible for the shaping of the genetic program (Mayr 1993, p. 94).

Gardner criticizes our “literal reading of Mayr”, implying that we have misunderstood Mayr’s intent. This is untenable, both because Mayr (1961, 1974, 1993) reiterated his stance over four decades, and because countless other commentators, including Watt, Haig, Beatty (1994), Dewsbury (1999), Ariew (2003), Thierry (2005), Amundson (2005), and others, endorse our reading. It is widely acknowledged that Mayr was ambiguous over the meaning of ultimate causation, and the resulting literature is confused as a consequence. Although Haig portrays us as suggesting Mayr uses ultimate in the historical rather than functional sense, we have always read Mayr as suggesting both. Mayr sees function and evolutionary history as connected, and indeed they are, as is ably illustrated by Gardner. However, unlike Gardner, we believe that problems often result from this stance, since natural selection of genetic variation is neither the only, nor a complete, explanation of the origin and evolution of (apparent) design. Linking function and evolution in a single term has had the unfortunate consequence of constraining the explanations that can be given for design features. It was for that reason that we endorsed Tinbergen’s (1963) four questions as “superior to Mayr’s… [because it]… clearly delineates function and evolutionary history” (Laland et al. 2012).

Gardner criticizes us for invoking other evolutionary processes (e.g. drift) as part of ultimate explanations, but his attribution of this “error” to our reciprocal causation stance is obviously not correct, since Dickins and Barton, who reject our position, make the same “error”. Given his emphasis on historical explanations, Mayr invites readers to conceive of a broader notion of “ultimate cause” than one tied to design, and characters brought to fixation through drift have an evolutionary cause too. Gardner quotes Ariew (2003) as saying: “it is difficult to imagine anyone accepting, say, genetic drift as a species of ultimate explanation”. In fact, the first part of Ariew’s sentence is actually a rejection of the term “ultimate explanation” (Ariew also rejects “ultimate cause”). Ariew recommends that these terms be replaced by “evolutionary explanation” since migration, mutation, recombination and drift “should be included as part of the conception that undergirds ‘ultimate’ explanations.” We can all agree that if the term ‘ultimate explanations’ is restricted to those characters exhibiting design then it is unlikely drift can explain them. Gardner’s complaint is with how Mayr cast “ultimate causes” and not with the accuracy of our portrayal of Mayr.

Given the confusion that “ultimate causation” now engenders, Haig recommends that the term be abandoned. We applaud this suggestion. There are perfectly adequate alternative terms that can be used to discuss these issues with far greater clarity (e.g. function, mechanism, evolutionary history). Haig follows many commentators in suggesting that Mayr coined the term “ultimate” causation at least partly for political reasons: “ultimate valorizes the evolutionary over the merely proximate”. Such valorization, if it were ever justified, has clearly become counterproductive.

## What aspects of proximate-ultimate causation should be rejected?

We have stressed that “Mayr’s concern that proximate and ultimate explanations should not be confused as alternatives remains valid and valuable” (Laland et al. 2012) and that “biologists will always require different answers to how and why questions” (Laland et al. 2011, p. 1516). In spite of this, both Dickins and Barton and Gardner interpret our message as a rejection of the value of distinguishing between proximate causes, function, and evolutionary history. Let us reiterate our position: we believe these distinctions are vital, including in cases where there is reciprocal causation, but that Mayr’s language is a hindrance to clarity precisely because it wraps together function and evolutionary history.

Dickins and Barton and Gardner are at pains to demonstrate that it is possible to explain cases with selective feedback in terms of proximate-ultimate causation, but this is not contested. In describing sexual selection we ourselves wrote:

The ultimate explanation for the male trait is the prior existence of the female preference, proximately manifest in peahen mate-choice decisions (Laland et al. 2011, p. 1512).

We believe that Dickins and Barton go too far with their suggestion that it is *impossible* to describe cases with selective feedback without recourse to ‘proximate’ and ‘ultimate causation’, or synonymous terms. As noted above, there are far better terms available that are less ambiguous and lack the dichotomy’s baggage. Neither are such terms precise synonyms of ultimate causation, because the latter has multiple meanings. However, there are more substantive issues at stake than disagreements over terminology.

The point we were making with our sexual selection example was that such cases do not fit with Mayr’s characterization of ontogenetic processes as *solely* proximate. Here, peahen preferences simultaneously act as (proximate) influences on mate-choice decisions *and* as (ultimate) sources of selection on peacock plumage. We further suggested that cases of reciprocal causation are now known to be far more extensive than they appeared to be in 1961, such that the claim that ontogenetic processes are also relevant to evolutionary accounts is a general claim, rather than germane to special cases. Thus our rejection of the proximate-ultimate dichotomy is a repudiation of Mayr’s equation of development with the solely proximate, and subsequent insistence that development is irrelevant to evolution.

Dickins and Barton seek to rewrite history by claiming that Mayr did not deny that development can affect evolution. Their position is at odds with all of the published historical analysis, which universally acknowledges Mayr’s insistence of the irrelevance of development to evolution, particularly later in his career (Gottlieb 1992; Gould 2002; Amundson 2005; Winsor 2006; Gilbert and Epel 2009), and both Watt and Calcott endorse our position. One of the most authoritative sources is the philosopher and historian Ron Amundson (2005), who writes:

Mayr never gives a hint of how it would be possible to relate development to evolution without committing the proximate-ultimate fallacy, so it is hard to resist the conclusion that Mayr believes that the irrelevance of development to evolution follows directly from the distinction itself (p. 223).

Amundson ends his analysis by concluding that satisfactory integration of evolution and development may require the rejection of the proximate-ultimate dichotomy (and other restrictive dichotomous terms). Mayr’s resistance to integrating evolution and development is well renowned. For instance, Mayr (1963, p. 609) is famous for asserting (wrongly, of course) that searching for homologous genes, or indeed other relevant features of development for evolution, would be futile (see Gould 2002, for discussion). Neither was Mayr alone in claiming evolution could, and should, be studied through population genetics alone. Dobzhansky (1951), and most of the other leading evolutionary biologists, took the same line for much of their careers:

Evolution is a change in the genetic composition of populations. The study of mechanisms of evolution falls within the province of population genetics.

However, Mayr’s position hindered the integration of evolution and development in a second, more-subtle, way too—by reinforcing the characterization of development as an unfolding of a genetic program (Gould 2002; West-Eberhard 2003; Amundson 2005). This implied that knowledge of genotype and selective environment was sufficient to describe evolutionary change. Mayr’s genetic “program” and “blueprint” metaphors are very much alive today, including in Dickins’ writings (e.g. Dickins and Rahman 2012). We discuss the issues of how best to characterize development, and evolutionary biology’s externalism (the view that the adaptations of organisms can be understood relative to the characteristics of external selective environments, Godfrey-Smith 1996), in detail below.

## How the proximate-ultimate causation dichotomy channels thinking

Gardner refrains from using the term ‘ultimate causation’, and instead speaks of ‘ultimate explanations’, presumably because he recognizes that this use of ‘causation’ would be dubious. Can any problems with the proximate-ultimate dichotomy be resolved merely by changing terms? Dickins and Barton maintain that since contemporary evolutionists know what they mean by proximate and ultimate explanations, there is no problem. We, of course, agree that much good work is done within the evolutionary sciences by recognizing that separate answers to how and why questions are needed, but the same good work could be done, and often is, using alternative terminology, without any of the negative ramifications of Mayr’s framework. The primary problem with the proximate-ultimate dichotomy is not that it is ambiguous, but that it channels thinking, leading to the neglect of alternative hypotheses.

Gardner points to both a logical and a formal connection between function and a history of selection. We accept his argument, based on Grafen’s (2002) mathematical analysis, that natural selection leads organisms to act as if maximizing their inclusive fitness. However, where strong adaptationism is coupled with genetic determinism and unidirectional models of causation it becomes problematical. It would be a mistake to assume that, because natural selection of genetic variation leads to design, that all semblance of design arises solely from natural selection of genetic variation. We foresee many characters that exhibit design features but are not biological adaptations (e.g. spandrels, exaptations, products of cultural evolution, as well as the appearance of design brought about through niche construction). Gould and Lewontin (1979) pointed out over three decades ago that features (e.g. spandrels) can possess design yet not be biological adaptations, and in spite of the progress made in the intervening period recent reviews have concluded “even in the presence of both functional data and evidence for selection from DNA sequence data, it is still difficult to construct strong arguments in favor of adaptation” (Nielsen 2009, p. 2487), and “the ‘adaptive’ designation may be premature and may lead to incorrect conclusions about the relationships between gene function and fitness” (Barrett and Hoekstra 2011, p. 767). Contrary to Gardner’s claim, we do see value in adaptationist thinking within biology, for instance, as a useful vehicle for generating hypotheses; however, it becomes a problem if it leads researchers to fail to consider viable alternative hypotheses. For instance, we don’t disagree with Gardner’s defense of Fisher’s modeling, but our point about Fisher’s emphasis on adaptive genetic variation with small effects is that Fisher, like most mainstream evolutionary biology from the 1930s to the present, prematurely dismissed saltationist and developmental explanations for evolutionary change. Fisher did this entirely by assumption. For instance, he wrote of the “*logical case* for rejecting the assumption that the direction of evolutionary change is governed by the direction in which mutations are taking place” (Fisher 1930, p. 17, our italics). We likewise worry that, by wrapping together function and evolutionary history, the ultimate causation conception leads researchers to think about evolutionary causality in linear terms, and to neglect alternative explanations for the appearance of design. We will illustrate our concern that the proximate-ultimate dichotomy blinkers thinking with two examples, one Gardner’s and the other from Dickins and Barton’s writings.

Gardner expresses apprehension over the “tangled mess of causation” that he envisages follows from our emphasis on reciprocal causation. This graphic phrasing is very instructive. Naturally, we agree that there is infinite regress of interlinked causal influences for any current event—if so inclined, researchers could trace causation back in time all the way to the big bang—but the suggestion that it is “not conducive to successful biological science” is a little overdramatic. The reciprocal causation stance is perfectly operational. It merely places the onus on researchers to make sensible judgments as to how far to trace back causality for the problem in hand. Evolutionary biologists do this all the time (as do scientists in many other fields). If reciprocal causation were genuinely crippling then there would be no theory of sexual selection or coevolution. In practice, researchers merely require a different kind of formulation, such as coupled equations, to accurately describe the evolutionary dynamics. The fact that these bodies of theory are progressive establishes that cases with reciprocal causation are not inherently problematic to study.

It is nonetheless fascinating and instructive that Gardner should view reciprocal causation as a problematical “tangled mess”. This seems to betray a fear that if evolutionary biologists were to let go of their unidirectional, externalist model of causation then chaos would ensue. But there is nothing to fear here. The ‘reciprocal causation’ stance requires researchers to make a judgment about where it is sensible to view causation starting. However, we regard this as a virtue, since it leaves researchers open to considering the full range of possibilities. For instance, a bout of sexual selection could start with a mutation generating variation in the trait, a novel environment eliciting variation in preference, with a pre-existing sensory bias, and so forth. Sorting between these alternative accounts is an empirically tractable issue, and researchers consider all these possibilities precisely because it is clear that causation is reciprocal.

Compare this open stance with Gardner’s (e.g. West et al. 2011) critique of an account of the evolution of cooperation through social norms (e.g. Boyd and Richerson 1985; Fehr and Fischbacher 2003; Gintis 2003; Henrich 2004). Gardner’s theoretical position constrains him to view this body of work as muddling proximate and ultimate causation, as from his standpoint social norms (for instance, to reward cooperators or punish defectors) can only be proximate causes. However, those criticised regularly make the distinction between proximate and ultimate causes in their own work (see, for instance, Fehr and Fischbacher 2003). We do not think either of the camps, both consisting of highly respected evolutionary thinkers, are making a schoolboy error; rather, they are separated by different models of causation. Gardner’s resistance to conceiving of the social norms explanation as a case of reciprocal causation left him and his coauthors unable to consider (or too quick to dismiss) the possibility that in this instance causation starts with plasticity in human cooperators, which generates selective feedback at either cultural or genetic levels. West et al. (2011) assert that imitation, punishment, and the satisfaction derived from these, are proximate answers to the question of why people cooperate, and complain that:

This does not solve the ultimate problem, because it does not answer why evolution should have produced a psychology or nervous system that mechanistically encourages (rewards) such punishment.

But where researchers are thinking in terms of reciprocal causation, it may do. Society-based institutions to reward cooperators and punish norm-violators will generate (natural or cultural) selection for tendencies to cooperate, and may modify (natural or cultural) selection on the pre-existing tendencies to reward/punish, and the satisfaction so derived. Of course, one can ask how the society-based cooperative norms, and/or predispositions to punish norm violators, came into existence in the first place, and answers to these questions push back the causal account further. Plausibly, cooperative norms piggy back on the prior existence of a sense of fairness that evolved through reciprocity or group selection, or an ancient tendency to retribution (Richerson and Boyd 2005; Richerson and Henrich 2012). However, the prior existence of such tendencies cannot be regarded as a complete “ultimate” explanation, since without the social norms to stabilize cooperation it does not evolve in this instance. Previous work by cultural evolution researchers has established that culture takes human populations down evolutionary pathways not available to non-cultural species, either by creating conditions that elicit established mechanisms (e.g. kinship, reciprocity) or via mechanisms not seen in other taxa (e.g. Henrich 2009), to generate an evolved psychology (tribal instincts, docility, shame applied to norms) that is entirely different from what can evolve through genes alone (Boyd and Richerson 1985; Henrich 2009; Chudek and Henrich 2011). Fehr, Boyd, Richerson and Henrich (personal communications) have confirmed that our characterization of their thinking about causality (in essence, that this is an instance of reciprocal causation) was correct, and that Gardner and his colleagues had misconceived their position.

The second illustration of how the proximate-ultimate dichotomy makes communication difficult can be found in Dickins and Barton’s treatment of developmental bias. In our articles we discussed cases like the repeated rapid adaptation of sticklebacks to post-glacial lakes through the loss of a pelvic girdle (Chan et al. 2010) and rapid adaptation and range expansion of the house finch in North America (Badyaev 2009), arguing that these examples, alongside many others, illustrate how developmental processes construct evolutionary pathways. In the first case, fast phenotypic change results from the elevated mutability of a major regulatory control gene, which, in a single step, generates an adaptive phenotype, and does so repeatedly in isolated populations, which are then subject to parallel (rather than convergent) evolution. In the second example, developmental processes respond to environmental challenges to generate functional, directional and coordinated suites of morphological and behavioural traits, in both parent and offspring (the latter through parental effects), which expose genetic variation to strong selection. This is perhaps the best-documented example of the Baldwin effect, a clear case in which (to coin West-Eberhard’s evocative phrase) “genes are followers, not leaders, in evolution”. Yet Dickins and Barton dismiss such cases as merely “lineage differences in the amount of proximate modular architecture there is for an overall phenotype” and “differences in how sensitive to environmental cues those proximate mechanisms are.” They conclude: “the proposal that natural selection and developmental bias are different explanations is a misunderstanding”.

Let us consider the differences between these two explanations (more accurately characterized as natural selection with and without developmental bias) for such examples more closely. Table 1 compares some general features of a conventional account with a developmental plasticity/bias explanation. To us the differences between these two classes of explanation are striking. For instance, in the standard account evolutionary change begins with genetic mutation, which generates phenotypic differences that are subject to selection. Conversely in the developmental plasticity/bias account phenotypic variation can also result from differential environmental induction, with genetic change following. In the standard account, genetic mutations (and novel phenotypes) are random with respect to direction, rate and location, typically disadvantageous, and appear in a single individual. Conversely, in the developmental plasticity/bias account, genetic mutations can be non-random with respect to rate and location, whilst novel phenotypes can be directional, functional, and may appear in multiple individuals. In the standard account, mutations vary from one population to the next, and are typically multiple and small in effect, with strong convergent selection in similar environmental conditions required to bring about similar phenotypes. In the developmental plasticity/bias account, the same mutation may appear in isolated populations, often associated with a suit of coordinated phenotypic changes, and is subject to parallel (that is, identical rather than convergent) selection. Further differences are given in the table.

**Table 1:** Differences between a conventional account and a developmental plasticity or developmental bias account of isolated populations adjusting to novel environmental conditions

H_1_: standard account	H_2_: developmental plasticity/bias account
1. Genetic mutation (plus recombination, migration) is the primary source of novel phenotypic variation. Genetic change precedes phenotypic change	Environmental induction is a major source of novel phenotypic variation with evolutionary potential. Genes may be followers, not leaders, in evolution (West-Eberhard 2003)
2. Mutations (and novel phenotypes) are random in direction and typically disadvantageous	Novel phenotypic variants may be directional and even functional
3. Isolated mutation (and novel phenotype) appears in a single individual	Novel phenotypic variants may be environmentally induced in multiple individuals
4. Character typically assumed to be based on many mutations of minor effect	Character may be product of major mutation in regulatory control gene, or major reorganization of developmental process
5. Random rate and location of genetic mutation	Non-random rate and location of genetic mutation (i.e. some variants produced more readily than others)
6. Mutations expected to vary across populations	Same mutation may appear in each population
7. Evolution via natural selection (in similar environments) is convergent	Evolution via natural selection is often parallel
8. Selection fashions and propagates adaptation	Developmental processes fashion adaptation then adaptive variants spread through selection and other mechanisms (e.g. learning, cultural inheritance)
9. Rapid phenotypic change attributed to strong selection	Rapid phenotypic change may result from the simultaneous induction and selection of functional phenotypes

Laid out in this manner, we believe most readers would recognize that that there are real and substantive differences between the standard and developmental plasticity/bias explanations. How then can Dickins and Barton deny this? Perhaps Dickins and Barton are unfamiliar with some aspects of the literature. It is hard to imagine that anyone conversant with the massive current interest amongst the evo-devo community in developmental systems as a source of evolutionary innovation (Gilbert et al. 1996; Arthur 2004; Minelli and Fusco 2004; Muller and Newman 2005a, b; Gilbert 2006), would characterize the issue of “the starting point of the explanation” (i.e. the origin of evolutionary innovation) as “somewhat trivial”, as Dickins and Barton do. Yet we think there is something more here: specifically, that the proximate-ultimate causation dichotomy encourages a unidirectional view of biological causation, where development is viewed as the outcome rather than the cause of evolution. In this respect we believe Gardner’s and Dickins and Barton’s stances are representative of a broader community of researchers (see e.g. Oyama et al. 2001; Keller 2010 for discussion).

We (like many other developmentally minded evolutionists, e.g. West-Eberhard 2003) believe that resistance to these ideas derives in part from implicit models of causation that can channel thinking on these topics, leading to the neglect of potentially important explanations. For instance, in their recent review of phenotypic plasticity’s impacts on speciation, where extensive evidence that plasticity is evolutionarily consequential was presented, Pfennig et al. (2010, p. 459) nonetheless conclude that “recent reviews of speciation generally fail to discuss phenotypic plasticity, indicating that workers in this field do not recognize a significant role for plasticity in speciation”. It is now well documented that there is considerable variation in mutation rates (e.g. Hodgkinson and Eyre-Walker 2011), but we suspect that the implications of this for developmental bias are rarely appreciated. In the particular case of pelvic reduction in sticklebacks, most evolutionary biologists would probably automatically reach for a counter-explanation of pre-existing standing genetic variation for rapid parallel evolution (e.g. Barrett and Schluter 2008), and this may well be tenable. However, the important point here is not that an explanation in terms of the elevated mutability of a key developmental control gene must be correct, but rather that it must be considered. Valid alternative explanations should not be ruled out on fallacious logical grounds.

## Additional problems with the proximate-ultimate causation dichotomy

Both Watt and Calcott draw attention to additional problems with the proximate-ultimate dichotomy. Watt writes that “Mayr’s dichotomy misrepresents the causal process of natural selection, and thus is entirely harmful”. He describes how it encourages neglect of Genotype–Phenotype–Environment (G–P–E) interactions that “are the causal sources of selection itself”. Ontogenetic processes are the immediate cause of natural selection. He criticizes the “amechanistic” stance of the MS, and emphasizes that to understand how natural selection operates researchers need to know about the mechanisms of development and how they interact with the ecological environment to generate fitness differences between genotypes. We couldn’t agree more, and applaud Watt’s pioneering empirical studies that demonstrate how developmental processes construct fitness differences between genotypes (Watt 1985, 2000, 2003; see also Badyaev 2009). Watt’s subtle analysis is a strong counter-perspective to Dickins and Barton, who seem to think that a rejection of the proximate-ultimate dichotomy implies a failure to distinguish between evolution and development. It is quite different to maintain that developmental processes shape G–P–E interactions, and that selection arises from a mismatch between these processes and the environment in which they function (Badyaev 2011), than to suggest that developmental processes *are* natural selection or evolution. Watt’s points are important, since it is easy to slip into regarding natural selection as a force, which externalizes it, leading to a neglect of how the organism itself affects its own fitness landscape (Endler 1986). We agree with Watt that “Mayr’s dichotomy distorts our view of natural selection, and must give way to more realistic, mechanistically inclusive approaches to the evolutionary process”. Indeed, we envisage this focus on the processes of development and how they interact with the internal and external environment to generate fitness differences between genotypes to be a core feature of an extended evolutionary synthesis.

Calcott shows another respect in which Mayr’s dichotomy is “too simple”, pointing out that there are questions that do not fit in either proximate or ultimate category. We think Calcott has a point when he says that Mayr’s scheme does not easily encompass lineage explanations. Lineage explanations were perhaps historically mainly used by paleontologists, but they are becoming increasingly common for developmental biologists that have data on genomic or developmental features of a few selected taxa. Thus, Calcott’s argument is another example of how researchers can get stuck in their own cause-and-context explanatory framework such that they struggle to see that there are other ways of thinking (or asking evolutionary questions). We think he is right to stress that this is an additional barrier to the integration of developmental and evolutionary biology.

## Clarification of ‘reciprocal causation’

Calcott asks for clarification of reciprocal causation, whilst Gardner writes “‘reciprocal causation’ is not conducive to successful biological science”, implying that he has misunderstood our usage of this term. ‘Reciprocal causation’ is not a theory, it is an entirely descriptive term. Reciprocal causation merely means that A is a cause of B and, subsequently, B is a cause of A. Of course, we are particularly interested here in biological systems and reciprocal causation is feature of evolving systems, for example, when the activities of organisms modify selective environments or modify the variants subject to selection, thereby influencing evolutionary trajectories. Reciprocal causation is also a feature of developing systems, where the organism, or features of it, modify developmental environments, be they internal or external, to shape the dynamic phenotype (Gilbert 2003). There is nothing mysterious or contentious about reciprocal causation.

While accepting that biological systems are reciprocally caused, Watt argues that at any point in time, causation is usually unidirectional. We accept that the reciprocal causal arrows need not always be operating simultaneously, and may occur in sequence. We view this as an empirical issue, to be evaluated on a case-by-case basis.

## The role of developmental processes in evolution

Where reciprocal causation is occurring, since it is the phenotype rather than the genotype that is the source of selection in response to which the biological system evolves, developmental processes potentially influence the evolutionary dynamics. Of course, whether researchers accept our claim that developmental processes play an important role in evolution depends very much on how they conceive of development, and on what evolutionary role they attribute to development.

In describing studies of sexual selection, coevolution and habitat selection we (Laland et al. 2012) wrote:

These studies capture a core structural feature of reciprocal causation—namely, selective feedback—but typically without truly embracing its full ramifications; specifically, without recognizing that this means that developmental processes can initiate and co-direct evolutionary outcomes.

Haig prima facie appears to deny that this role for development in evolution does go unrecognized. He writes:

Do developmental mechanisms play a role? Does organismal evolution alter the environment and subsequent selection? Few would dissent from an affirmative answer to either question.

However, closer inspection establishes that we are in accord, at least with respect to what is contentious. Haig’s claim (personal communication) should be read as an empirical statement. He seeks to establish what it is that lies at the heart of debates over the role of development in evolution by clarifying what is accepted by all parties. He is saying, and we agree, that no-one disputes that organisms modify environments, and no-one disputes that as a result of this subsequent selection is changed. This is tantamount to our claim that the selective feedback implicit in cases of reciprocal causation is uncontroversial.

Few would deny that development plays *a* role—but what role precisely? Related to this, Haig’s final sentence reads:

There is nothing in this view that denies an important role for development in evolutionary processes, nor that a recursive relationship exists among genotypes, phenotypes, and environments.

Once again, interpretation of this sentence hangs critically on what the “important role” is. Consider two possible interpretations. Is the ‘important role of development’ to translate genetic variation into phenotypes (or extended phenotypes) with differential fitness, under the direction of information encoded in the genome, leading to a change in the genetic composition of the population (and potentially also the environment) through selection, thereby ‘resetting the clock’ for selection to act again on a revised gene pool, in an endless recursion? Conceivably, there might be some recognition of passive developmental constraints here too, such that development can be seen as playing a role, in the same sense of, say, population size, by affecting evolutionary outcomes. But, on this view, development is not a creative or directing evolutionary process; rather it is a hindrance to selection. If that is the correct reading, then we would agree that most evolutionists would accept that development does at least this, but we would emphasize that it is not a strong claim. In principle, even an extreme genetic determinist, who believed that every aspect of the phenotype was fully prescribed by genetic information, could, by this reasoning, endorse Haig’s statement. Perhaps it is this kind of reasoning that leads Dickins and Barton to write:

What niche construction boils down to is that adaptation occurs in an historical context: what comes before influences what comes next.

Dickins’ earlier writings explicitly claim that “epigenetic variation is under genetic control” and strongly imply that he believes that learning and culture are too (Dickins and Rahman 2012). Perhaps if one believed that every evolutionarily significant aspect of the phenotype is pre-specified by genetic information, niche construction might appear to amount to little more than this. Indeed, were it to be established that developmental systems do not introduce novelty and do not impose any directionality onto the action of natural selection, such cases of reciprocal causation would reduce to repeated bouts of unidirectional causation. But we would argue it is also not what the debate is about.

We see a major evolutionary debate over whether (as Gould, West-Eberhard, Muller, Lewontin, Odling-Smee et al. etc. would claim) developmental processes share with natural selection some of the responsibility for the direction and rate of evolution. This relates to the second interpretation of Haig’s final sentence. This reading might envisage some additional “important role for development”, for instance, in introducing evolutionarily significant novelty into phenotype design space, or systematically biasing the action of selection by shaping selective environments, or controlling which genetic variants are exposed to selection, or if variation in evolvability related to developmental plasticity explains taxonomic differences in diversity more effectively than variation in selective environments. We are not convinced that most evolutionists would accept that developmental processes play these additional, more creative, roles in evolution (among our commentaries, Dickins and Barton certainly do not). Such roles must be regarded as currently contentious, and it is here that the debate should lie. This reading of Haig would require us to take issue with his claim. The proximate-ultimate conceptualization of causation may not, in any absolute sense, deny these roles for development in evolution, but its association with a unidirectional model of causation and a blueprint metaphor for development makes it more difficult for researchers to conceive of these roles being plausible. Recursive relationships among genotypes, phenotypes and environments of a sort are recognized, but not recursive relationships in which development really changes very much. Development remains “left out” of the synthesis, and natural selection retains sole responsibility for explaining both adaptive design and diversity. Haig (personal communication) confirms that his meaning is closer to the first reading than the second, which leaves us in agreement over what the debate is about, even if we take different positions on the issues.

## Characterizing phenotypic plasticity

Mayr’s (and others) characterization of development as an unfolding on a “genetic program” has left an unfortunate legacy, as noted by many authors (Lewontin 1983; Oyama 1985; Weber and Depew 2001; Keller 2010; Bateson and Gluckman 2011). For example, Noble writes

The genome is sometimes described as a program that directs the creation and behaviour of all other biological processes in an organism. But this is not a fact. It is a metaphor. It is also an unrealistic and unhelpful one (Noble 2006, p. 51).

The problem with the program metaphor is that it feeds into the popular dogma that genes cause proteins, which cause cells, which cause tissues and organs, and so forth, and in this (unidirectional) way the phenotype is created by the genotype. It creates the false impression that developmental processes are *prescribed* by genetic information. In reality, reciprocal causation operates throughout development too. For instance, proteins are required for the cellular machinery to read the genes that produce proteins. In multiple ways, causation flows from higher levels of organismal organization back to the genes. For instance, there are many different means of reading the genes (many splice variants) and how such genes are read depends in part on the cellular environment. There are higher-level triggers of cell signalling and higher-level control of gene expression. Moreover, a lot of what proteins do—how they fold, combine and interact—is not dependent on instructions from genes but on the chemistry of self-assembling complex biological systems (Noble 2006). There is no overarching program that directs this assembly; rather it is a process in which the developing organism shapes its own developmental trajectory, constantly responding to environmental inputs, and constantly altering those environments (Oyama et al. 2001; Gilbert 2006; Gilbert and Epel 2009). For these reasons, it is a distortion to characterize the phenotype as “under genetic control” or developmental processes as tightly regulated by naturally selected genes. Development is not prescribed, it is *constructive*. (We deploy the term ‘constructive’ to characterize development, sensu Oyama et al. (2001), not to valorize it but to emphasize that the organism is genuinely constructing its own development).

Natural selection explains the capability for plastic responses but not the content (e.g. it explains why people have the ability to learn a language, but not why a particular individual speaks French). There are, of course, genetic influences on how organisms respond to environmental inputs, but this is a long way from the level of genetic control envisaged by Dickins and Rahman (2012). The significance of a more open, flexible conception of development is that the developmental process itself becomes a source of novel, yet functional and coordinated, phenotypic variation. (Conversely, were development to be prescribed then no adaptive phenotypic novelty could ever emerge throughout development).

As Watt emphasises, not all phenotypic plasticity is accurately portrayed as genetically controlled “switches” that regulate plastic responses. Watt points out that the term “phenotypic plasticity” covers a lot of disparate phenomena, and distinguishes between several classes of it. Perhaps some simple forms might usefully be characterized as adaptive switches that respond to differential environmental inputs in predictable ways, whilst other more complicated cases, such as involving learning and cultural transmission, cannot. Here selection has favoured additional information-gathering and storing organism-environment interactions, precisely because the optimal phenotype cannot be pre-specified a priori. We think Watt is right, and that work is required to establish to what extent these different forms of plasticity have evolutionary potential. However, we note that the enhanced evolvability facilitated by even comparatively simple polyphenisms has been shown to affect macro-evolutionary patterns (Pfennig et al. 2010), leaving us open to the view that all forms of plasticity may be potentially evolutionarily significant.

## An extended evolutionary synthesis

Amongst the various lobbies for extensions to the modern synthesis we see certain themes beginning to crystallize. Two such themes are an emphasis on reciprocal causation and on a constructive conceptualization of development. Put together, these two ideas allow developmental processes to generate novel phenotypes with evolutionary potential and to co-direct the action of selection through developmental bias and niche construction. These ideas further allow for an expanded conception of inheritance that recognizes that additional inheritance systems will affect evolutionary dynamics and outcomes. Whilst the various lobbies for change in evolutionary biology draw from many disparate fields, we nonetheless see a coherence to their arguments.

Yet, at the same time, it is probably fair to say that these various lobbies currently more resemble a disorganized protest movement than a viable alternative government. There is not yet a clear statement as to precisely what any extended evolutionary synthesis (EES) entails, what its core assumptions are, and how they differ from the modern synthesis. In this light, we think it is not unreasonable of Dickins and Barton to assert:

to create the paradigm shift that Laland et al. seek, this theory would have to make novel predictions that biologists do not already make or conceive of.

We find such talk of ‘paradigm shifts’ unhelpful. The change that we would like to see within evolutionary biology is potentially compatible with the vast bulk of the MS, and our calls, and those of others who endorse the EES, are for reformation not revolution in evolutionary thinking. However, as Pigliucci and Muller (2010, p. 4) state, “being consistent with the MS is not at all the same as being part of the MS!”, and we strongly believe that a broader conceptualization of the processes that direct evolutionary change would prove enormously fruitful. As an illustration of this reasoning, we will end our response by rising to Dickins and Barton’s challenge in a more modest way, and consider what novel predictions arise from the examples of developmental plasticity that we have discussed.

A quick glance at Table 1 immediately reveals that there are many such predictions that follow from the EES position, since each row can be recast as a pair of alternative hypotheses. For instance, the MS predicts that genetic mutations (and hence novel phenotypes) will be random in direction and typically disadvantageous, whereas the EES predicts that novel phenotypic variants will frequently be directional and functional. The MS expects isolated mutations (with novel phenotypes) will occur in a single individual, whereas the EES predicts that novel, evolutionarily consequential, phenotypic variants will frequently be environmentally induced in multiple individuals. In isolated populations exposed to similar environmental conditions, the MS predicts different genetic variations will arise, whereas the EES predicts that the same genes will mutate again and again. Further novel predictions can be deduced from the table. (We emphasize that the EES does not deny the explanations or hypotheses of the MS, it merely wishes to extend these explanations). These are empirically tractable issues, and there is considerable current interest amongst the evolutionary biology community in exploring them. It is early days, and far more data are required to draw any strong conclusions. However, we would argue that there are already sufficient data consistent with the expectations of the EES (West-Eberhard 2003; Odling-Smee et al. 2003; Muller 2007; Pfennig et al. 2010; Moczek et al. 2011) for it to merit serious consideration. If our articles on causation serve to encourage researchers to consider alternative ways of thinking and thereby investigate these important issues then we will consider those articles a success.
